# Analysis of association of *MEF2C*, *SOST* and *JAG1* genes with bone mineral density in Mexican-Mestizo postmenopausal women

**DOI:** 10.1186/1471-2474-15-400

**Published:** 2014-11-28

**Authors:** Rafael Velázquez-Cruz, Rogelio F Jiménez-Ortega, Alma Y Parra-Torres, Manuel Castillejos-López, Nelly Patiño, Manuel Quiterio, Teresa Villarreal-Molina, Jorge Salmerón

**Affiliations:** Laboratorio de Genómica del Metabolismo Óseo, Instituto Nacional de Medicina Genómica, Mexico City, Mexico; Unidad de Vigilancia Epidemiológica Hospitalaria, Instituto Nacional de Enfermedades Respiratorias, Mexico City, Mexico; Subdirección de Desarrollo de Aplicaciones Clínicas, Instituto Nacional de Medicina Genómica, Mexico City, Mexico; Unidad de Investigación Epidemiológica y en Servicios de Salud, Instituto Mexicano del Seguro Social, Cuernavaca, Morelos Mexico; Laboratorio de Enfermedades Cardiovasculares, Instituto Nacional de Medicina Genómica, Mexico City, Mexico; Centro de Investigación en Salud Poblacional del Instituto Nacional de Salud Pública, Cuernavaca, Morelos Mexico

**Keywords:** Association study, *MEF2C*, *SOST*, *JAG1*, Genes, Mexican Women

## Abstract

**Background:**

Osteoporosis, a disease characterized by low bone mineral density (BMD), is an important health problem in Mexico. BMD is a highly heritable trait, with heritability estimates of 50-85%. Several candidate genes have been evaluated to identify those involved in BMD variation and the etiology of osteoporosis. This study investigated the possible association of single-nucleotide polymorphisms (SNPs) in the *MEF2C*, *SOST* and *JAG1* genes with bone mineral density (BMD) variation in postmenopausal Mexican-Mestizo women.

**Methods:**

Four hundred unrelated postmenopausal women were included in the study. Risk factors were recorded and BMD was measured in total hip, femoral neck and lumbar spine using dual-energy X-ray absorptiometry. In an initial stage, a total of twenty-five SNPs within or near *SOST* gene and seven SNPs in the *JAG1* gene were genotyped using a GoldenGate assay. In a second stage, three *MEF2C* gene SNPs were also genotyped and *SOST* and *JAG1* gene variants were validated. Real time PCR and TaqMan probes were used for genotyping.

**Results:**

Linear regression analyses adjusted by age, body mass index and ancestry estimates, showed that five SNPs in the *SOST* gene were significantly associated with BMD in total hip and femoral neck but not lumbar spine. The lowest *p* value was 0.0012, well below the multiple–test significance threshold (*p* = 0.009), with mean effect size of -0.027 SD per risk allele. We did not find significant associations between BMD and *MEF2C*/*JAG1* gene variants [rs1366594 “A” allele: β = 0.001 (95% CI -0.016; 0.017), *P* = 0.938; rs2273061 “G” allele: β = 0.007 (95% CI -0.007; 0.023), *p* = 0.409].

**Conclusions:**

*SOST* polymorphisms may contribute to total hip and femoral neck BMD variation in Mexican postmenopausal women. Together, these and prior findings suggest that this gene may contribute to BMD variation across populations of diverse ancestry.

**Electronic supplementary material:**

The online version of this article (doi:10.1186/1471-2474-15-400) contains supplementary material, which is available to authorized users.

## Background

Osteoporosis (OP) is a common skeletal disease characterized by reduced bone mass, microarchitecture deterioration, and a reduction in bone mineral density (BMD), leading to increased bone fragility and susceptibility to fracture [1]. OP is a serious public health problem in Mexico causing up to 30,000 osteoporotic fractures a year, and an annual health service expenditure of about $97 million in 2006 [2]. While multiple risk factors influence the pathogenesis of osteoporosis, genetic factors play an important role in BMD variation. Heritability of this trait is high, as twin and family studies show that genetic factors account for approximately 50% to 85% of BMD [3].

Several candidate genes have been evaluated to assess their role in BMD variation and the etiology of osteoporosis, mainly in populations of European and Asian ancestry [4–6]. Research has focused on the Wnt/β-catenin signaling pathway, which is known to play a major role in bone remodeling [7]. The *SOST* (*sclerostin*) gene encodes the sclerostin protein, which binds to LRP5/6 co-receptors and antagonizes Wnt/b-catenin signaling in both osteocytes and osteoblasts [8, 9]. Several *SOST* single nucleotide polymorphisms (SNPs) have been associated with BMD variation [6, 10–12]. Moreover, the *MEF2C* (*myocyte enhancer factor 2C*) gene, another member of the Wnt-signaling pathway [13], is known to play an important role in determining bone density and mediating inflammatory effects in bone [14] and Mef2C is the main transcriptional factor responsible for ECR5-dependent Sost expression in the adult skeleton of mice [15]. Within this gene, rs1366594 has been associated with Femoral Neck (FN) BMD in Europeans, and with Total Hip (TH) BMD in populations from East-Asia [4, 5]; while rs119510131, was found to be significantly associated with forearm BMD in the meta-analysis of 6,584 individuals of European and Mexican American descent [16].

In another pathway, Jagged-1 has been found to induce human mesenchymal stem cell (hMSC) osteoblast differentiation through canonical Notch signaling [17]. A genome-wide association study (GWAS) reported that the rs2273061 polymorphism within the *JAG1* (*jagged 1*) gene was associated with low Lumbar Spine (LS) BMD, FN BMD, and with osteoporotic fractures in subjects of European and Asian descent [18].

To date, most studies examining the relationship of *SOST*, *MEF2C* and *JAG1* polymorphisms with BMD variation have been performed in populations of European and Asian ancestry [4–6, 10–12, 18]. The aim of this study was to analyze the possible association of polymorphisms in these genes with BMD variation in Mexican-Mestizo postmenopausal women.

## Methods

### Study population

The study group included only women born in Mexico whose parents and grandparents identified themselves as Mexican Mestizos. This study was performed as part of the third stage of a previously described, long-term cohort study [19, 20]. A total of 400 unrelated postmenopausal women over 45 years of age, without spontaneous menses for at least 1 year, attending the Instituto Mexicano del Seguro Social (IMSS) located in Cuernavaca, Morelos State, were recruited. Exclusion criteria were a history of bone, metabolic or endocrine disease, history of oophorectomy prior to 45 years of age, menopause prior to 40 years of age, or use of medication interfering with bone metabolism. Data on demographic characteristics and information regarding smoking status, menopausal status, estrogen use, medical history, and use of medication were obtained from self-administered questionnaires. The study was approved by the IMSS Research Ethics Committee and all participants provided signed informed consent. Blood samples were obtained and stored at 4°C until use. Genomic DNA was extracted from the peripheral blood of all participants, using a commercial isolation kit (QIAGEN systems Inc., Valencia, CA), according to the manufacturer’s instructions.

### Measurement of bone mineral density

BMD at the lumbar spine (L2-4), femoral neck, and total hip were assessed using a dual X-ray absorptiometry (DXA) Lunar DPX NT instrument (Lunar Radiation Corp., Madison WI). BMD was calculated from bone mineral content (g) and bone area (cm^2^), and then expressed as g/cm^2^.

### Genotyping and single nucleotide polymorphism selection

In an initial phase of the study, a GoldenGate platform with a total of 384 SNPs was used to test for associations with BMD, including twenty-five *SOST* and seven *JAG1* SNPs [21]. These SNPs were selected using the following criteria: minor allele frequency (MAF) >5% in Caucasians, and polymorphisms previously associated with BMD in candidate gene association studies, genome wide association studies (GWAS) and meta-analyses [4, 6, 10–12, 18]. Genotype data were extracted for further analysis.

In the present study, we extended the analysis to seek other possible associations between SNPs and BMD variation in another member of the Wnt signaling pathway, the *MEF2C* gene, analyzing three SNPs (rs12521522, rs11951031 and rs13665949). Furthermore, we genotyped five *SOST* SNPs (rs9911277, rs4793018, rs1983490, rs1881107 and rs4792909), and three *JAG1* SNPs (rs2273061, rs6040061 and rs2235811) in 30% of randomly selected samples. Genotyping of these eleven SNPs was performed using commercial predesigned TaqMan Probes (Applied Biosystems, Foster City, CA, USA) in a StepOne Plus RT PCR system. Information on the parental frequencies and genotype data for a panel of 96 Ancestry Informative Markers (AIMs) was extracted from the initial phase genotypes, in order to control for the effect of false associations due to population stratification.

### Statistical analyses

All data from the population in the study are showed as mean ± SD (standard deviation) for quantitative variables and absolute and relative frequencies for qualitative variables. Hardy-Weinberg equilibrium was tested for each SNP using the standard χ^2^ test. Linear regression analyses were used in order to test for associations between BMD and genotype using an additive genetic model adjusting for age and body mass index (BMI). Ancestry from principal component analysis (PCA) was estimated using the smartpca program in the Eigensoft 3.0 package [22], and ancestry estimates were included as confounding factors for correction of population stratification. All statistical analyses were performed with Statistical Package for Social Sciences software (SPSS 20.0; SPSS Inc.; Chicago, IL, USA). *P*-values <0.05 were considered statistically significant. Linkage disequilibrium (LD) and haplotype frequencies were estimated using Haploview 4.2 [23]. Statistical power was calculated with Quanto 1.1 software, for a significance level of 0.05 and MAF of 5% in 400 postmenopausal women with a minimal power of 80% to detect differences in BMD, under an additive model. The significance threshold after multiple test correction for each gene was estimated using the single nucleotide spectral decomposition software (SNPSpD) [24]. This approach can be applied to obtain an effective number of independent marker loci (Meff). The application of SNPSpD to our nine SNP sample set gave a Meff of 5.48 SNPs, leading to a significance threshold of 0.05/5.48 = 0.009.

## Results

The quality control (QC) criteria for subject and SNP genotyping for the initial phase have been previously described [21]. Briefly, data from 14 individuals whose DNA did not genotype successfully were excluded and 11 more participants were discarded from subsequent analysis because their African ancestry exceeded the mean reported for the Mexican mestizo population [25]. In the second phase, the overall genotype call rate was 98%, and the genotypes showed 100% reproducibility with those obtained in the initial phase. Two *MEF2C* SNPs (rs12521522 and rs11951031) were not observed in this sample of Mexican women, and were thus excluded from the analysis.

The general characteristics of the participating women are presented in Table 1. Mean age was 62.25 ± 9.11 years, and a mean BMI was 28.08 ± 4.76 kg/m^2^. Mean bone parameters were within normal range, although mean LS BMD was 0.990 ± 0.151 (mild osteopenia), which is expected given the age of study participants.

**Table 1:** **Demographic characteristics and BMD of postmenopausal Mexican Mestizo women**

Variable	Mean (SD)
Age (Yr)	62.25 (9.11)
Height (cm)	152.98 (5.74)
Weight (Kg)	65.73 (11.74)
BMI^a^ (Kg/m^2^)	28.08 (4.76)
BMD^b^ total hip (g/cm^2^)	0.929 (0.133)
BMD^b^ femoral neck (g/cm^2^)	0.880 (0.125)
BMD^b^ lumbar spine (g/cm^2^)	0.990 (0.151)
Age of Menarche	12.94 (1.55)
Number of children	3.12 (2.13)
Duration of breastfeeding (months)	15.90 (19.86)
Years since menopause	17.84 (11.59)
Estrogen remplacement therapy^c^	110 (26.00)
Tobacco use^c^	137 (34.33)
Alcohol intake^c^	45 (11.26)
Carbonated beverage consumption^c^	315 (78.75)

*N* = 400 Postmenopausal women.

^*a*^ *BMI* = *Body Mass Index*.

^*b*^ *BMD* = *Bone Mineral Density*.

^c^n (%); n, number of women; SD, standard deviation.

In the initial stage, twenty-five *SOST* and seven *JAG1* SNPs were tested for single-marker allelic association, adjusting for age, body mass index (BMI) and ancestry estimates. In the second phase, five *SOST* and three *JAG1* SNPs showing significant associations with BMD were further analyzed. SNP details are described in Table 2. Genotype and allele frequency distributions of all SNPs did not differ from Hardy-Weinberg equilibrium (Table 3).

**Table 2:** **Information on the SNPs analyzed in this study**

Gene	SNP	Allele	Position	Location in the gene
***MEF2C***	rs1366594	C/A	5:88376061	Intergenic
***SOST***	rs9911277	G/A	17:41780484	Intergenic
	rs4793018	C/T	17:41781069	Intergenic
	rs1983490	G/A	17:41782957	Intergenic
	rs1881107	A/G	17:41786076	Intergenic
	rs4792909	G/T	17:41798824	Intergenic
***JAG1***	rs2273061	A/G	20:10639543	Intron variant
	rs6040061	A/C	20:10640306	Intron variant
	rs2235811	C/T	20:10644158	Intron variant

**Table 3:** **Genotype**/**allele frequencies and Hardy**-**Weinberg equilibrium** (**HWE**) ***P*** **values for the analyzed SNPs**

^a^SNP reference number	Genotype	N	%	^b^HWE
**rs1366594** ***MEF2C***	C/C	186	46.6	p = 0.30
	C/A	166	41.6	
	A/A	47	11.8	
Allele frequency	C	538	67.4	
	A	260	32.6	
**rs9911277** ***SOST***	G/G	113	28.6	p = 0.70
	G/A	193	48.9	
	A/A	89	22.5	
Allele frequency	G	419	53.0	
	A	371	47.0	
**rs4793018** ***SOST***	C/C	117	29.5	p = 0.58
	C/T	192	48.4	
	T/T	88	22.2	
Allele frequency	C	426	53.7	
	T	368	46.3	
**rs1983490** ***SOST***	G/G	121	30.3	p = 0.55
	G/A	192	48.1	
	A/A	86	21.6	
Allele frequency	G	434	54.4	
	A	364	45.6	
**rs1881107** ***SOST***	A/A	122	30.7	p = 0.43
	A/G	189	47.6	
	G/G	86	21.7	
Allele frequency	A	433	54.5	
	G	361	45.5	
**rs4792909** ***SOST***	G/G	116	29.2	p = 0.27
	G/T	187	47.1	
	T/T	94	23.7	
Allele frequency	G	419	52.8	
	T	375	47.2	
**rs2273061** ***JAG1***	A/A	132	33.2	p = 0.43
	A/G	201	50.5	
	G/G	65	16.3	
Allele frequency	A	465	58.4	
	G	331	41.6	
**rs6040061** ***JAG1***	A/A	147	37.1	p = 0.21
	A/C	198	50.0	
	C/C	51	12.9	
Allele frequency	A	492	62.1	
	C	300	37.9	
**rs2235811** ***JAG1***	C/C	151	38.1	p = 0.43
	C/T	193	48.7	
	T/T	52	13.1	
Allele frequency	C	495	62.5	
	T	297	37.5	

^*a*^ *SNP* = *single nucleotide polymorphism*; ^*b*^ *HWE* = *Hardy*-*Weinberg Equilibrium*.

In the second phase, linear regression analyses adjusted by age, BMI, and ancestry estimates showed that *SOST* gene SNPs rs9911277, rs4793018, rs1983490, rs1881107 and rs4792909 were significantly associated with TH-BMD and NF-BMD (mean effect size of -0.027 per risk allele; lowest *p* value = 0.0012) but not with LS-BMD (Table 4). *MEF2C* gene rs1366594 failed to show a significant association with any BMD estimation (p value range 0.938 - 0.542). On the other hand, *JAG1* gene SNPs rs2235811 and rs2275811 showed a trend to association with FN-BMD and LS-BMD (*p* = 0.051 and 0.089, respectively); well above the multiple test-significance threshold estimated at 0.009. After correction for multiple testing, only the association of BMD with *SOST* polymorphisms remained significant.

**Table 4:** **Association of** ***MEF2C***, ***SOST*** **and** ***JAG1*** **polymorphisms with BMD in postmenopausal Mexican**-**women**

	Gene	dbSNP	β (95% IC)^d^	*p*_*adjust*_^*e*^
**BMD**-**TH** ^**a**^ **(g/** **cm** ^**2**^ **)**	*MEF2C*	rs1366594	0.001 (-0.016; 0.017)	0.938
	*SOST*	rs9911277	-0.031 (-0.046; -0.016)	0.000072
		rs4793018	-0.027 (-0.043; -0.012)	0.00035
		rs1983490	-0.027 (-0.042; -0.012)	0.00040
		rs1881107	-0.027 (-0.042; -0.011)	0.00055
		rs4792909	-0.025 (-0.040; -0.010)	0.0012
	*JAG1*	rs2273061	0.007 (-0.009; 0.023)	0.409
		rs6040061	0.009 (-0.007; 0.026)	0.280
		rs2235811	0.011 (-0.005; 0.027)	0.189
**BMD**-**FN** ^**b**^ **(g/** **cm** ^**2**^ **)**	*MEF2C*	rs1366594	0.004 (-0.011; 0.019)	0.638
	*SOST*	rs9911277	-0.029 (-0.043; -0.015)	0.000048
		rs4793018	-0.026 (-0.040; -0.012)	0.00024
		rs1983490	-0.027 (-0.041; -0.013)	0.00018
		rs1881107	-0.026 (-0.040; -0.012)	0.00027
		rs4792909	-0.025 (-0.039; -0.011)	0.00053
	*JAG1*	rs2273061	0.007 (-0.008; 0.022)	0.331
		rs6040061	0.013 (-0.003; 0.028)	0.109
		rs2235811	0.013 (-0.002; 0.028)	0.089
**BMD**-**LS** ^**c**^ **(g/** **cm** ^**2**^ **)**	*MEF2C*	rs1366594	0.007 (-0.015; 0.028)	0.542
	*SOST*	rs9911277	-0.006 (-0.027; 0.014)	0.539
		rs4793018	-0.007 (-0.027; 0.013)	0.498
		rs1983490	-0.007 (-0.027; 0.014)	0.514
		rs1881107	-0.003 (-0.023; 0.018)	0.806
		rs4792909	-0.002 (-0.022; 0.019)	0.865
	*JAG1*	rs2273061	0.021 (0.000; 0.042)	0.051
		rs6040061	0.008 (-0.014; 0.030)	0.484
		rs2235811	0.007 (-0.015; 0.028)	0.557

^a^BMD-TH = Total hip bone mineral density.

^b^BMD-FN = Femoral neck bone mineral density.

^c^BMD-LS = Lumbar spine bone mineral density.

^d^Data are shown for the additive model.

^*e*^ *p*-*adjust* values were adjusted by age, BMI, and ancestry estimates.

**Figure 1:**
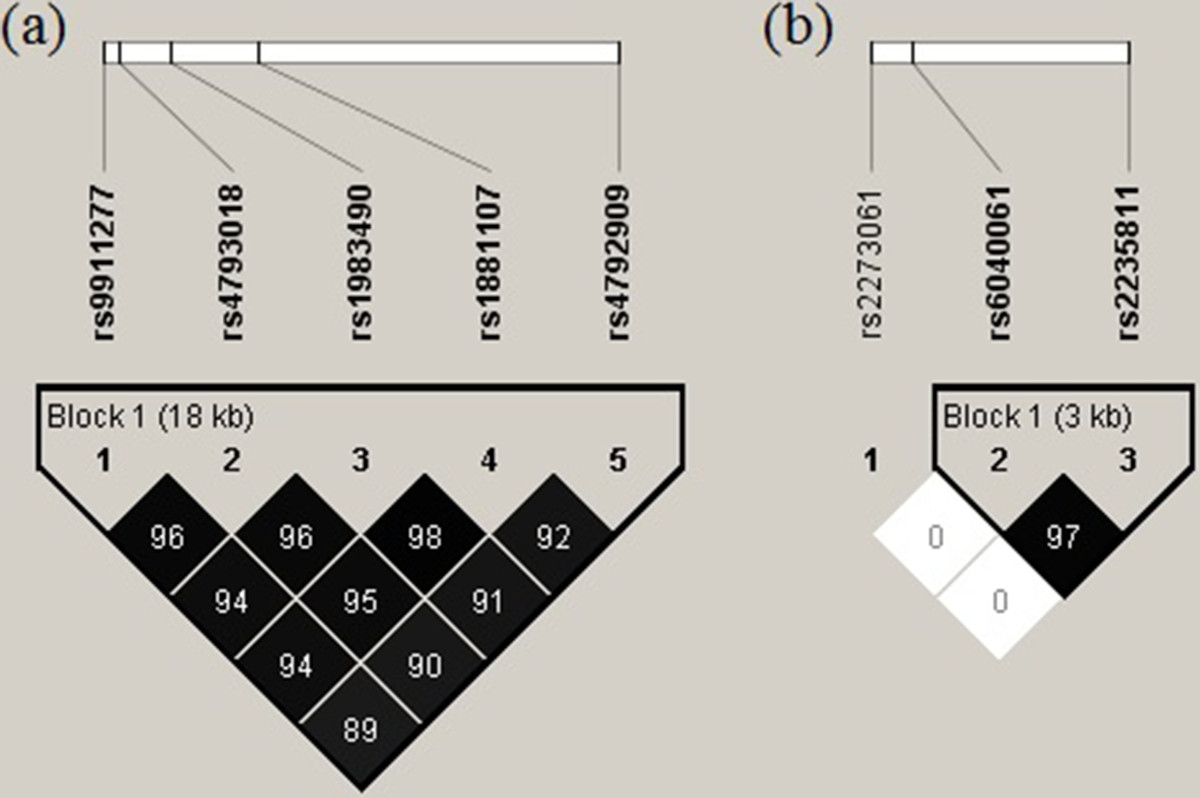
**Location and pair**-**wise linkage disequilibrium values of** ***SOST*** **and** ***JAG1*** **polymorphisms in Mexican postmenopausal women.** Darker color indicates higher LD and lighter color indicates less LD. Values of the pair-wise (r2) are shown to *SOST* **(a)** and *JAG1* **(b)** genes.

Linkage disequilibrium analysis revealed significant relationships among the *SOST* gene polymorphisms, identifying a single high LD block (pair-wise r^2^ values >0.89; Figure 1). Significant LD was observed across the gene, and we inferred the existence of two main haplotypes: ACAC and CACA, with frequencies of 0.49 and 0.48, respectively.

## Discussion

OP constitutes a serious and increasing public health problem in the Mexican population [2]. To date, GWAS and candidate gene studies have revealed 70 genetic loci associated with BMD variation, but these studies have been performed mostly in populations of European and Asian ancestry [5, 6]. In the present study, we analyzed the association of polymorphisms of the *MEF2C*, *SOST* and *JAG1* genes with BMD variation in postmenopausal Mexican-mestizo women.

We found that SNPs of the *SOST* gene were associated with total hip and femoral neck, but not with lumbar spine BMD. These results are consistent with recent genome-wide association studies [4–6, 11], and may be explained at least partly by a lower heritability for BMD in the femoral neck site. This is supported by a previous study reporting that hip BMD of grandmothers is better predictor of hip BMD in mothers (beta = 0.46); while maternal LS BMD was less predictive of LS BMD in grandchildren (beta = 0.30) [19].

Together, our findings support the association of Wnt signaling pathway gene polymorphisms with BMD variation in the Mexican-mestizo population [21]. SOST is a known Wnt signaling inhibitor, which acts suppressing LRP function. *In vitro*, SOST inhibits osteoblast development and is expressed primarily in osteocytes [26]. *SOST* polymorphisms have been associated with BMD at different skeletal sites and with osteoporotic fractures in various populations [10–12]. Our present finding of a SOST-BMD association in the Mexican-mestizo population is in agreement with a similar association found by Estrada *et al*. [6] and Richards *et al*. [11] in postmenopausal women of European ancestry.

The MAFs of *SOST* gene SNPs rs9911277, rs4793018, rs1983490, and rs1881107 observed in the present study were very similar to those reported in the HapMap in individuals of Mexican ancestry of Los Angeles (0.45 to 0.47), but were less frequent than in European populations (MAF: 0.35 to 0.38). On the other hand, the rs4792909 “G” allele, most widely reported and associated with fracture risk in a large meta-analysis, was less frequent in our population (0.53) than in Europeans (0.62) [6], but very similar to that observed in a Los Angeles population of Mexican ancestry (0.53), (http://hapmap.ncbi.nlm.nih.gov).

Conversely, we did not find the associations between specific *MEF2C* and *JAG1* SNPs and BMD variation previously identified in populations of European and Asian ancestry [4, 5, 18]. The *MEF2C* rs1366594 “A” allele was not associated with BMD variation in the present study, and was less frequent in Mexican postmenopausal women (0.33) than in Europeans (0.55), East-Asians (0.42) and Han Chinese individuals (0.40) [4, 5, 27]. Moreover, the *MEF2C* rs11951031 previously associated with forearm BMD in Europeans and individuals of Mexican-American descent was not informative in this sample of Mexican menopausal women. This may be partially explained by our sample size being too small to detect a risk allele with a frequency of 0.06 [16].

Similarly, we failed to observe associations of *JAG1* polymorphisms with BMD variation. *JAG1* rs2273061 was initially found to be associated with BMD variation (LS and FN) in a GWAS performed in Hong Kong Chinese individuals, and this association was afterwards confirmed in several European populations [18]. However, this SNP was not associated with BMD variation in a Mexican-Mestizo cohort [28], in consistency with our present observations. Moreover, the *JAG1* rs2273062 allelic and genotypic frequencies reported here are virtually identical to those described previously in Mexicans [28], and the “G” allele frequency (0.42) was similar to that reported in Europeans (0.40), but higher than in the population of Hong Kong (0.31) [18]. The rs2235811 and rs6040061 MAFs observed in Mexican postmenopausal women were similar to those published in the dbSNP database for a population of Asian descent (http://www.ncbi.nlm.nih.gov/SNP/). However, it is notable that although Kung et al. reported that rs2273061, rs6040061 and rs2235811 are in high LD in individuals of both Chinese (r^2^ > 0.9) and European (r^2^ > 0.7, CEU) descent [18], in our population rs2273061 was clearly not in LD with the other *JAG1* polymorphisms (Figure 1). This may explain why rs2273061 was not associated with BMD in this study. Further studies including other *JAG1* SNPs will define whether this gene is associated with BMD or not in the Mexican population.

The Mexican population is admixed and has a complex genetic structure including European, Native American and a small proportion of African genes [29, 30]. Spurious association signals produced by differences in ancestral background can be a confounding factor in genetic association studies [31, 32]. Since population stratification may be a source of spurious associations, we used the first and second principal component from PCA, described previously [21] to account for population stratification. Our results suggest that population stratification was most likely not a confounding factor in this study.

Our study has some limitations. First, *MEF2C*, *JAG1* and *SOST* SNPS were selected based on previous reports from European and Asian populations, and thus other SNPs within the same genes may contribute to BMD variation in Mexican-mestizos. Second, the failure to observe associations of *MEF2C* and *JAG1* SNPs with BMD in our population may be due to the small sample size and reduced statistical power of the study [33]. Although our sample size (400 individuals) was large enough to identify the effect of *SOST* gene variants on BMD, in order to replicate the previously observed association of *MEF2C* rs1366594, considering a MAF of 0.33 and an effect size of 0.08 (in standard deviations of BMD), a total of 2,769 samples would be needed to achieve 80% statistical power to detect a significant association. The statistical power of the present study to find a significant association with this SNP was only 19%. Similarly, we would require 6,909 samples to obtain adequate statistical power to detect an association of *JAG1* rs2273061 with BMD variation, considering a MAF of 0.42 and an effect size of 0.048 SDs. Thus, even the combined results of the present study with those of Rojano-Mejía et al., who genotyped the rs2273061 in 750 Mexican individuals, would not reach adequate statistical power to replicate the association of this SNP with BMD. These results should be interpreted with caution, and should be considered as uncertain rather than null associations. Additional studies in larger samples are required to understand the role of *MEF2C* and *JAG1* gene variation in BMD variation in the Mexican population.

## Conclusions

In conclusion, our results suggest that *SOST* polymorphisms contribute to total hip and femoral neck BMD variation in postmenopausal Mexican-mestizo women. Taken together, these and other previously reported findings in the Mexican population suggest that variation in genes located in the Wnt pathway may be important contributors to BMD variation in the Mexican-mestizo population. Further investigation is required to understand interactions of genes located in this pathway with other factors, such as hormones and nutrition.

## Authors’ original submitted files for images

Below are the links to the authors’ original submitted files for images. Authors’ original file for figure 1
