# Supplementary figures and images for: Analysis of association of MEF2C, SOST and JAG1 genes with bone mineral density in Mexican-Mestizo postmenopausal women
Source: BMC Musculoskelet Disord. 2014 Nov 28;15:400. doi: 10.1186/1471-2474-15-400 (PMC4258010; doi:10.1186/1471-2474-15-400)

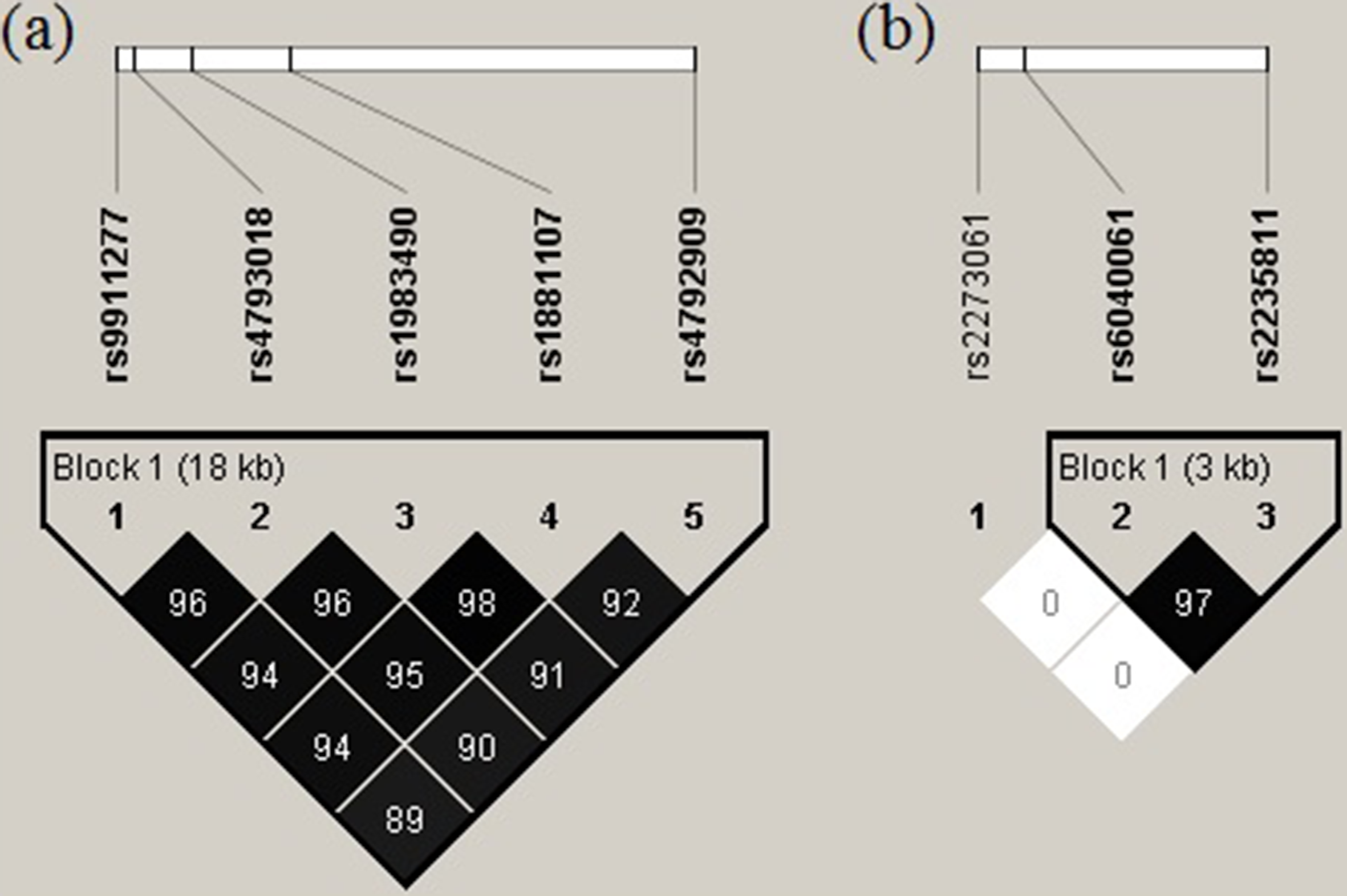

Supplement: Supplementary file 1 — Authors’ original file for figure 1 [file 12891_2014_2341_MOESM1_ESM.tif]
